# Herpes Simplex Virus Encephalitis With Cerebellar Infarction and Cortical Laminar Necrosis

**DOI:** 10.7759/cureus.102993

**Published:** 2026-02-04

**Authors:** Drashti S Parekh, Munir M Rajpura

**Affiliations:** 1 Neurology, Pramukhswami Medical College, Anand, IND; 2 Internal Medicine, Gujarat Medical Education and Research Society (GMERS) Medical College and Hospital, Sola, Ahmedabad, IND

**Keywords:** acute vertigo, cerebellar infarction, cortical laminar necrosis, diffusion-weighted mri, herpes simplex encephalitis, seizures and ataxia, vestibulocerebellar syndrome, viral encephalitis

## Abstract

Herpes simplex virus (HSV) encephalitis typically involves the temporal lobes, and cerebellar involvement with stroke and cortical laminar necrosis (CLN) is rare. A 40-year-old man with no prior comorbidities presented with 10 days of positional vertigo, vomiting and intermittent fever. Initial otorhinolaryngology assessment showed bilateral nystagmus and a positive fistula test, and he was treated as having a peripheral vestibular disorder. He re-presented with persistent vertigo and new confusion; on admission, he was febrile, hypertensive and ataxic, and within 24 hours, he developed worsening encephalopathy, focal deficits and generalised tonic-clonic seizures. Cerebrospinal fluid (CSF) analysis demonstrated lymphocytic pleocytosis, elevated protein, normal glucose, raised opening pressure and red blood cells, and polymerase chain reaction (PCR) for HSV DNA was positive, confirming HSV encephalitis. Brain magnetic resonance imaging (MRI) revealed an acute infarction in the right greater than the left cerebellar hemisphere with corresponding diffusion restriction on diffusion-weighted and apparent diffusion coefficient sequences. Follow-up imaging in the subacute phase showed curvilinear T1 hyperintensities along the cerebellar cortical ribbon consistent with CLN. The patient was treated with intravenous acyclovir, high-dose corticosteroids, antiepileptic drugs, osmotherapy, blood pressure control and supportive care. Over a three-week admission, his sensorium normalised, and seizures ceased, but truncal and appendicular ataxia persisted. At early outpatient review, he was ambulant with a cane, with mild residual truncal ataxia and no further seizures. This case highlights that HSV encephalitis can present with an acute vestibulocerebellar syndrome and be complicated by cerebellar infarction and CLN and emphasises the need for early neuroimaging and lumbar puncture in febrile patients with ‘peripheral’ vertigo.

## Introduction

Herpes simplex virus (HSV) encephalitis is the most common cause of sporadic fatal viral encephalitis in adults, with an incidence of approximately 2-4 cases per million per year and a marked predilection for the limbic and temporal lobes [1,2]. Untreated disease carries a mortality rate exceeding 70%, which has fallen to around 20% with the introduction of early acyclovir therapy, although many survivors have persistent neurological sequelae [1,3]. Typical manifestations include fever, headache, altered behaviour or consciousness, focal neurological deficits and seizures [1-3]. Neuroimaging usually demonstrates the asymmetric involvement of the medial temporal and inferior frontal lobes, while cerebellar involvement is distinctly unusual [2].

Cerebrovascular complications of HSV infection are increasingly recognised. Both haemorrhagic and ischaemic strokes have been described, probably related to a viral vasculitis or virus-induced vasculopathy affecting intracranial arteries [4]. Most reported cases involve supratentorial vessels, and posterior fossa infarction in HSV encephalitis is rare. In addition, severe metabolic or ischaemic insults to the cortex may lead to cortical laminar necrosis (CLN), which refers to selective neuronal necrosis affecting cortical laminae and typically manifests as gyriform cortical hyperintensity and cortical enhancement on magnetic resonance imaging (MRI) following ischaemic, hypoxic or metabolic injury.

CLN is a pathological pattern of selective neuronal death in the cortical ribbon that appears as gyriform T1 hyperintensity in the subacute phase on magnetic resonance imaging (MRI) [5,6]. CLN has been reported after global hypoxia, prolonged status epilepticus, hypoglycaemia and large territorial infarction, but it is infrequently described in association with HSV encephalitis and almost never in the cerebellar cortex [5-8].

We report a case of polymerase chain reaction (PCR)-confirmed HSV encephalitis in a previously healthy man, manifesting with an acute vestibulocerebellar syndrome, bilateral cerebellar infarction and cerebellar CLN. The case highlights important diagnostic considerations when evaluating acute vertigo with systemic features and illustrates the intersection of viral encephalitis, stroke and cortical necrosis.

## Case presentation

A 40-year-old right-handed man with no known comorbidities presented to the emergency department with a 10-day history of dizziness, repeated vomiting and intermittent low-grade fever. The dizziness was described as brief episodes of unsteadiness and a sensation of imbalance, occurring several times a day and precipitated by changes in head position. There was no initial history of headache, visual disturbance, ear pain, hearing loss or recent trauma.

He had been evaluated by the otorhinolaryngology service three days before admission. Examination at that time revealed spontaneous bilateral horizontal nystagmus and a positive fistula test. The external auditory canals and tympanic membranes were normal, with no otorrhoea, and bedside hearing tests suggested preserved hearing. A peripheral vestibular disorder was considered most likely, and he was treated symptomatically with betahistine, prochlorperazine and antiemetics.

Because symptoms persisted and his family noted new confusion, he presented to the emergency department. On arrival, his temperature was 38.5°C, pulse 104 beats/minutes, blood pressure 150/110 mmHg and oxygen saturation 98% on room air. He appeared drowsy but arousable, with a Glasgow Coma Scale score of 13/15 (E3 V4 M6). Neck stiffness was mild. Cranial nerve examination showed gaze-evoked horizontal nystagmus bilaterally, more prominent with rightward gaze; there was no facial weakness or ophthalmoplegia. Motor examination showed normal bulk and power in all four limbs, but finger-nose testing revealed dysmetria on the right, and heel-knee-shin testing demonstrated bilateral limb ataxia. Deep tendon reflexes were symmetric, and plantar responses were flexor. He was markedly unsteady when attempting to stand, requiring two-person assistance.

The presence of persistent vertigo, gait instability and associated focal neurological findings raised concern for a central aetiology rather than peripheral vestibular pathology.

Within the first 24 hours of admission, his mental status deteriorated. He became stuporous and experienced two generalised tonic-clonic seizures lasting approximately 1-2 minutes each. After the second seizure, there was a transient left-sided arm drift and right lower facial weakness. He was transferred to a high-dependency unit, loaded with intravenous levetiracetam (40 mg/kg) and commenced on maintenance therapy (1 g twice daily). Initial routine blood investigations showed mild leucocytosis and normal serum electrolytes and liver and renal function. A non-contrast cranial computed tomography scan was unremarkable.

**Figure 1:**
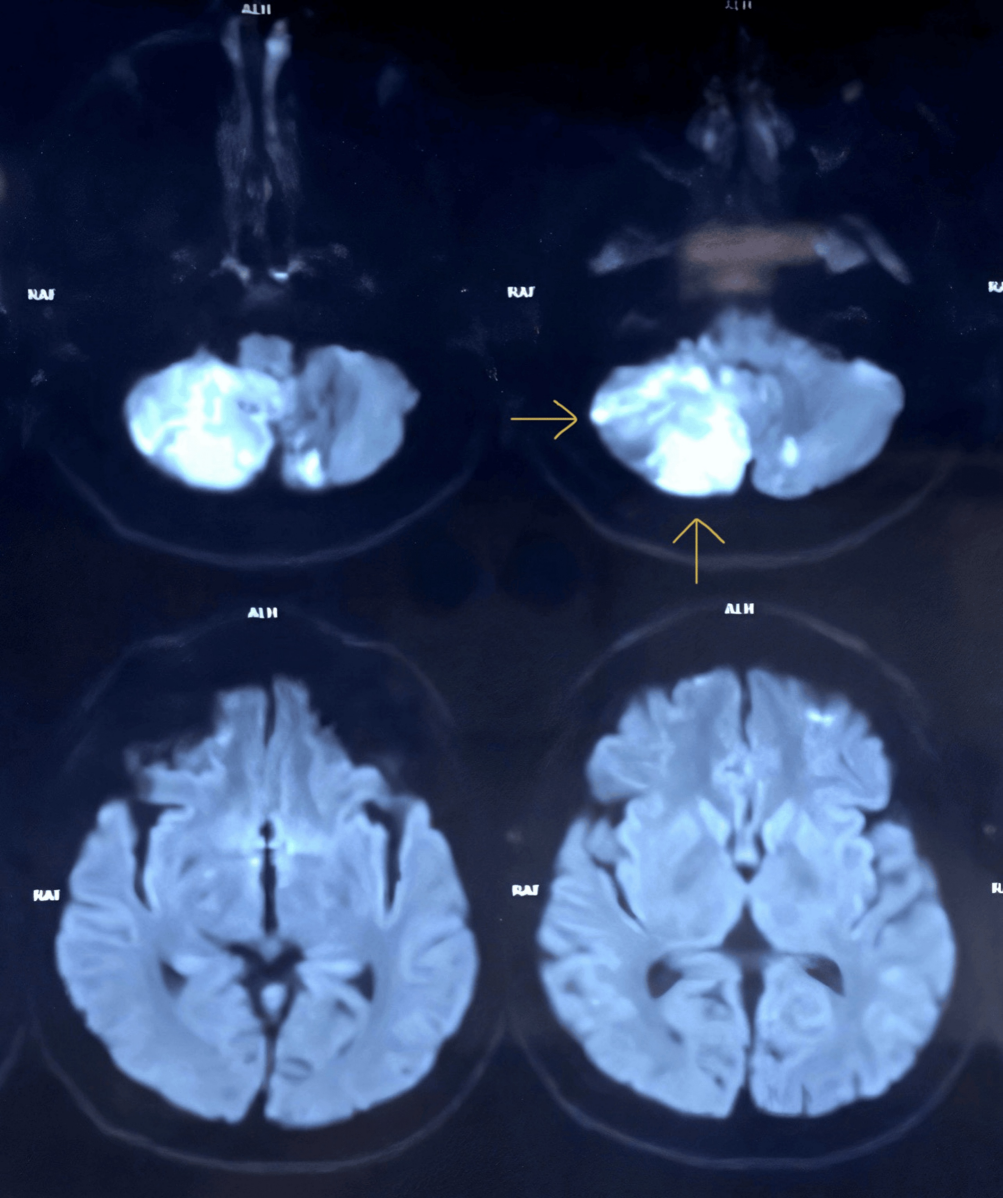
Diffusion-weighted MRI showing acute infarction in the right greater than the left cerebellar hemisphere. Axial diffusion-weighted MRI showing marked hyperintensity in the right cerebellar hemisphere with a small contralateral focus in the left (arrows), consistent with acute ischaemic infarction. MRI: magnetic resonance imaging

**Figure 2:**
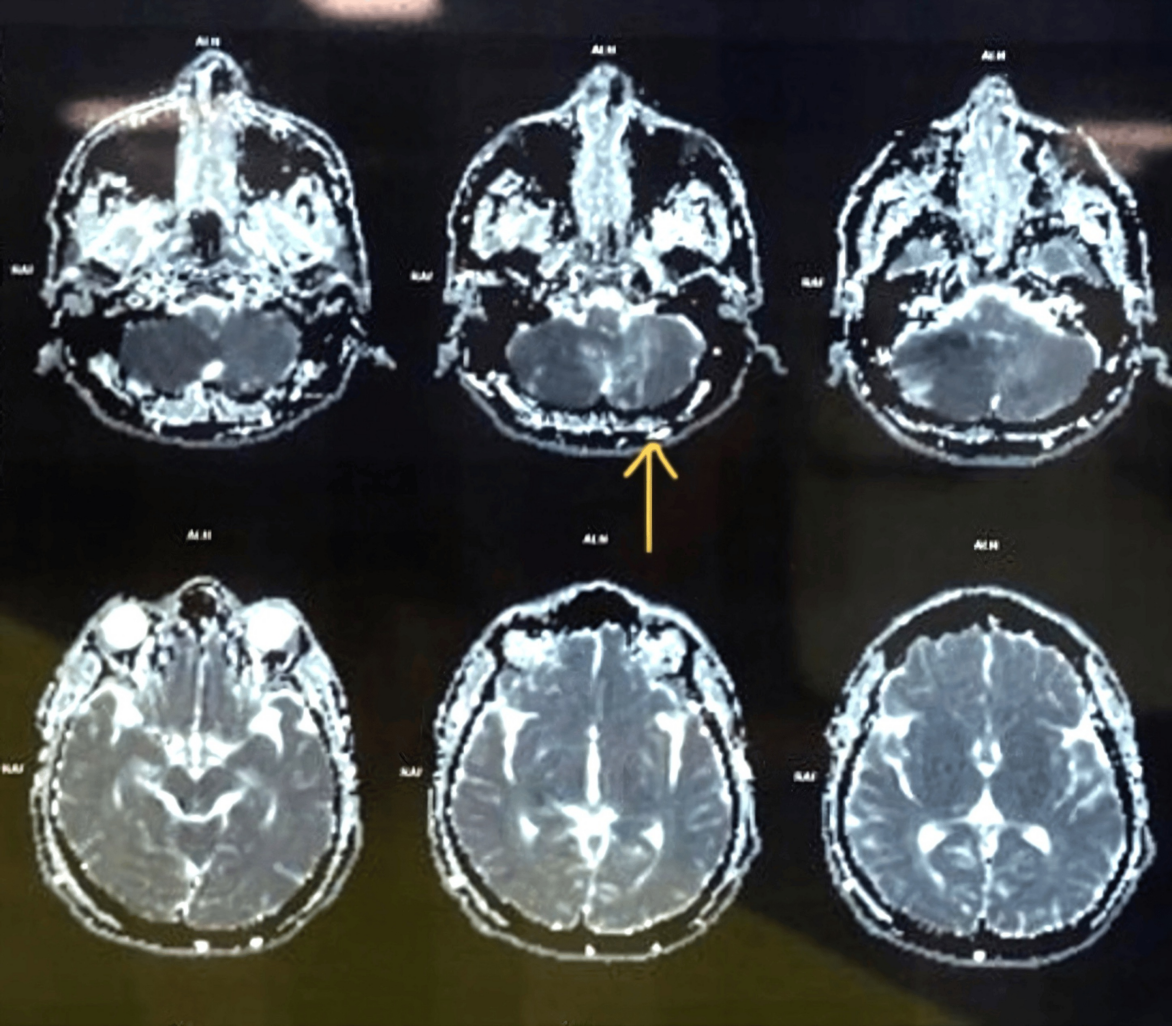
Apparent diffusion coefficient (ADC) map corresponding to Figure 1. Axial ADC images showing low signal in the right and left cerebellar hemispheres at the sites of diffusion restriction, confirming acute ischaemic infarction.

**Figure 3:**
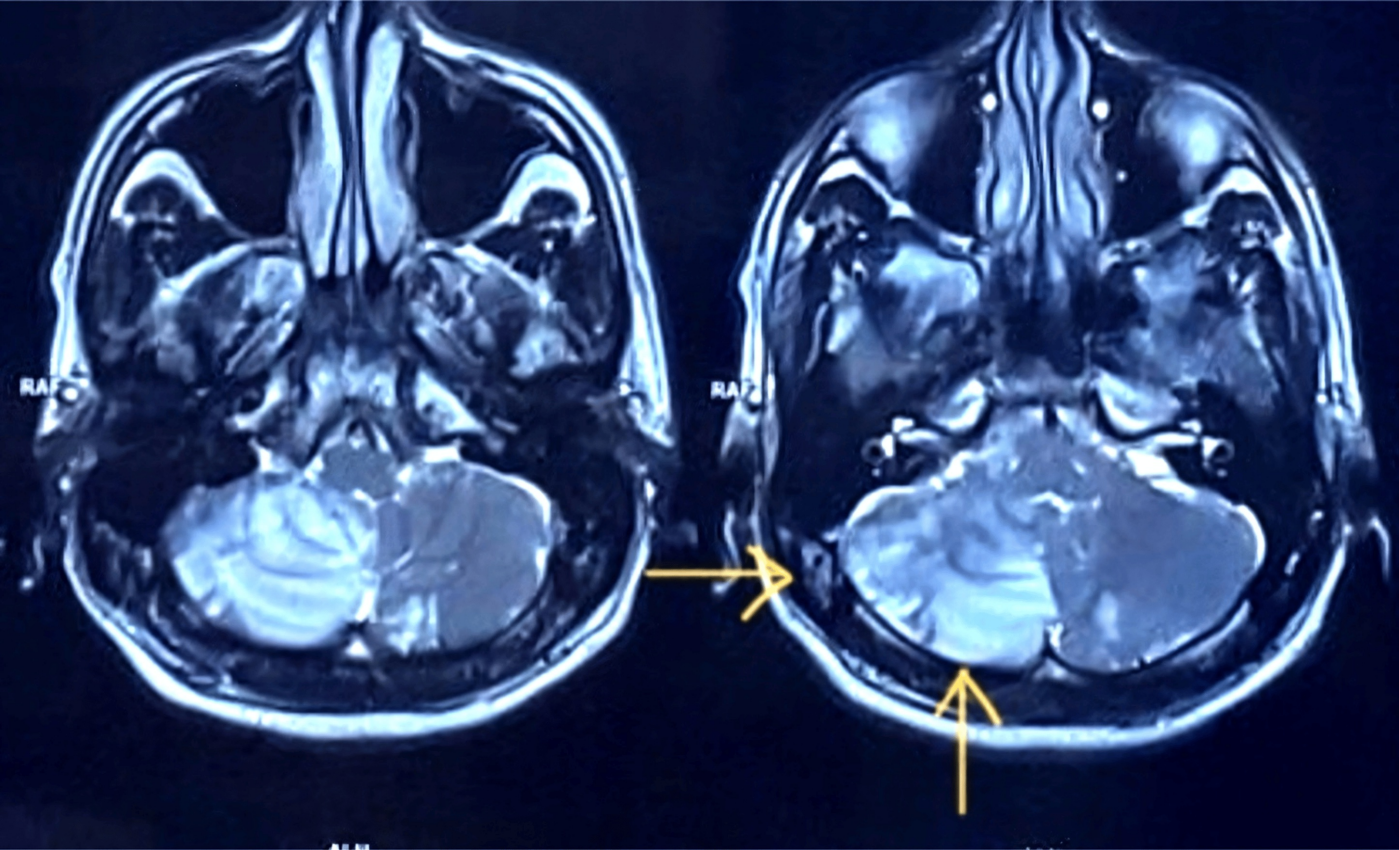
T2-weighted/FLAIR MRI showing oedema associated with bilateral cerebellar infarcts. Axial T2-weighted and FLAIR images demonstrating the hyperintensity and swelling of the right greater than the left cerebellar hemispheres, without haemorrhage or hydrocephalus. FLAIR, fluid-attenuated inversion recovery; MRI, magnetic resonance imaging

An MRI of the brain performed urgently demonstrated diffusion restriction in the right cerebellar hemisphere and a smaller focus in the left cerebellar hemisphere on diffusion-weighted imaging (Figure 1), with corresponding low signal on apparent diffusion coefficient maps (Figure 2), consistent with acute ischaemic infarction. T2-weighted and fluid-attenuated inversion recovery (FLAIR) sequences showed hyperintensity and swelling in these regions (Figure 3), without haemorrhage. The supratentorial brain parenchyma appeared normal, and there was no hydrocephalus.

Lumbar puncture was performed to investigate suspected meningoencephalitis. Cerebrospinal fluid (CSF) opening pressure was 27 cm H₂O. Analysis revealed 90 white blood cells/mm³ (lymphocytic predominance), elevated protein (120 mg/dL) and normal glucose relative to serum. Red blood cells were present. Gram stain and bacterial cultures were negative. Based on the clinical picture of fever, encephalopathy, seizures and this CSF profile, empirical intravenous acyclovir (10 mg/kg every eight hours) was initiated. PCR for HSV DNA subsequently returned positive, confirming HSV encephalitis.

These findings were consistent with viral encephalitis, and HSV polymerase chain reaction subsequently returned positive.

Over the next several days, the patient required intubation for airway protection due to fluctuating consciousness. He developed recurrent seizures, controlled with the escalation of antiepileptic therapy, including phenytoin. Given the bilateral cerebellar infarcts and concern for posterior fossa swelling, high-dose intravenous dexamethasone 1 g twice daily was administered for two days, followed by a tapering course, and mannitol was given intermittently to treat suspected raised intracranial pressure. Blood pressure was controlled with intravenous labetalol and subsequently oral amlodipine. Broad-spectrum antibiotics were discontinued once bacterial infection was excluded.

**Figure 4:**
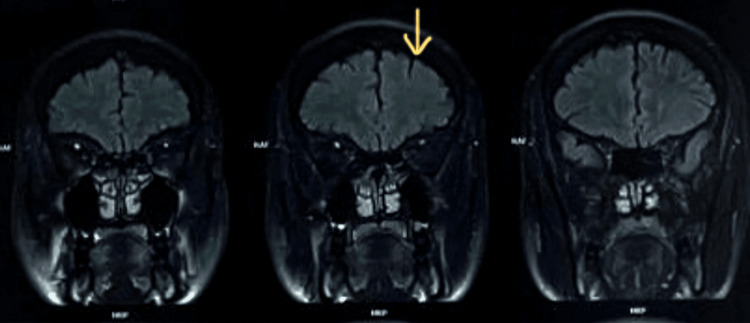
Subacute MRI demonstrating cerebellar cortical laminar necrosis. Coronal/axial T1-weighted images showing curvilinear hyperintensity along the cortical surface of the cerebellar hemispheres, more prominent on the right, in keeping with cortical laminar necrosis. MRI: magnetic resonance imaging

A repeat MRI study in the subacute phase showed evolving cerebellar infarction and new curvilinear hyperintense signal along the cortical surface of the cerebellar hemispheres on T1-weighted images (Figure 4), more prominent on the right, in keeping with cortical laminar necrosis. There were also early cerebral cortical atrophic changes. No large-vessel occlusion was identified on magnetic resonance (MR) angiography.

By the end of the third week of admission, the patient’s sensorium had normalised, and no further seizures occurred. He remained ataxic, with significant truncal instability and right-sided limb dysmetria, but muscle strength was full, and there was no residual paresis. He was discharged on oral levetiracetam 1 g twice daily, amlodipine 5 mg once daily and a tapering dose of oral corticosteroids, with instructions to complete a 21-day course of acyclovir.

At an outpatient review approximately one month after discharge, he reported marked improvement in dizziness and gait. He could walk independently for short distances but preferred a walking aid outdoors. Neurological examination showed mild residual truncal ataxia and minimal right-sided limb dysmetria; nystagmus was present only at extreme lateral gaze. There had been no further seizures. He had returned to light clerical work and was independent in basic activities of daily living. Longer-term follow-up and repeat MRI were not available.

## Discussion

This case illustrates several uncommon aspects of HSV encephalitis. First, the initial presentation was dominated by vestibulocerebellar symptoms, leading to an early working diagnosis of peripheral vertigo. Second, the course was complicated by bilateral cerebellar infarction, and third, subacute imaging demonstrated cerebellar cortical laminar necrosis (CLN). Taken together, these features highlight the capacity of HSV to cause both encephalitic and vascular injury in the posterior fossa and to trigger severe cortical metabolic damage.

HSV encephalitis most often affects the temporal and frontal lobes, presumably due to viral spread along trigeminal or olfactory pathways [1,2]. Cerebellar involvement is rare; a small number of cases of HSV cerebellitis have been reported, typically with subacute dizziness, ataxia and headache, sometimes in the absence of classical temporal lobe abnormalities on MRI [9-11]. Our patient similarly presented with vertigo and gait ataxia, but the presence of fever and altered mental status should raise the early suspicion of a central cause even when peripheral signs are present. This case underscores the need for urgent neuroimaging and lumbar puncture in patients with presumed peripheral vestibular disorders who develop systemic features or encephalopathy.

The differential diagnoses considered included ischaemic infarction, autoimmune or paraneoplastic encephalitis and metabolic or toxic encephalopathy. Ischaemic stroke was considered, given the cerebellar involvement; however, the gyriform cortical pattern, temporal progression of imaging findings and virologic confirmation supported an alternative aetiology. Autoimmune encephalitis was also considered, but the imaging characteristics and clinical course were more consistent with an infectious process. Metabolic and toxic causes were excluded based on the clinical context and available laboratory evaluation.

Cerebrovascular complications of HSV infection are increasingly recognised. A systematic review identified both ischaemic and haemorrhagic strokes occurring in association with HSV encephalitis, likely mediated by a necrotising vasculitis or immune-mediated vasculopathy of cerebral arteries [4]. Although most reported infarcts are supratentorial, posterior circulation involvement has been described, including medullary and cerebellar infarction [12]. In our patient, diffusion restriction and low apparent diffusion coefficient confined to the cerebellar hemispheres indicated genuine ischaemic infarction rather than purely inflammatory oedema. The absence of significant atherosclerotic risk factors, normal cardiac evaluation and a negative thrombophilia screen favour HSV-associated small-vessel vasculopathy and possibly transient haemodynamic compromise during seizures as contributors to his cerebellar strokes.

Cortical laminar necrosis represents a characteristic radiological end point of severe cortical injury. Histologically, it reflects the pannecrosis of neurons, glia and microvasculature in specific cortical layers, with a relative sparing of deeper white matter [5,6]. On MRI, CLN usually appears as a thin gyriform line of T1 hyperintensity, sometimes with corresponding T2/FLAIR signal change or contrast enhancement, emerging days to weeks after the inciting event [5,6]. Reported causes include prolonged global cerebral hypoxia, large territorial infarction, hypoglycaemia and long-standing focal status epilepticus [5-8]. In encephalitis, CLN is less frequently documented, but seizures and focal cortical inflammation provide a plausible mechanism. Our patient experienced recurrent seizures and bilateral cerebellar infarcts in the context of HSV infection, which together likely created a profound energy crisis in the cerebellar cortex, culminating in CLN. The presence of cerebellar rather than supratentorial CLN is unusual and emphasises that this process can occur in any cortical region subjected to sufficient metabolic stress.

The mainstay of therapy in HSV encephalitis is the early administration of intravenous acyclovir [1-3]. Guidelines recommend 10 mg/kg every eight hours for 14-21 days in immunocompetent adults [1,3]. Delay in initiating acyclovir is associated with worse outcomes. Our patient received acyclovir promptly once herpes simplex virus encephalitis was suspected, which likely contributed to his favourable outcome. The role of corticosteroids remains controversial; some observational data suggest that adjunctive steroids may reduce cerebral oedema and improve short-term neurological recovery without clearly increasing adverse events, whereas randomised data are limited [13,14]. In this case, substantial posterior fossa oedema and the risk of brainstem compression provided a rationale for short-term high-dose dexamethasone, similar to the approach reported in other cases of HSV cerebellitis with mass effect [9-11]. Seizure control is also critical, as status epilepticus independently contributes to neuronal injury and may promote laminar necrosis [7,8]. Combination antiepileptic therapy was required in our patient but ultimately achieved durable seizure freedom.

Prognosis after HSV encephalitis is variable. Despite treatment, many survivors exhibit residual cognitive, behavioural or motor deficits, and late unprovoked seizures are common [1,3]. Our patient’s outcome, survival with persistent but improving ataxia and no early seizure recurrence, can be considered favourable given the severity of his illness and the presence of cerebellar infarcts and CLN. Early rehabilitation and balance training are likely to have contributed to his functional gains. Longer-term follow-up would be valuable to document the evolution of his deficits and neuroimaging changes; however, this was not available.

This case adds to the small body of literature describing atypical HSV cerebellitis and HSV-associated stroke and is, to our knowledge, one of the very few reports of cerebellar CLN in this context. It reinforces the importance of considering HSV encephalitis in adults with acute vertigo accompanied by fever or encephalopathy and recognising that viral encephalitis can be complicated by ischaemic infarction and laminar necrosis.

Limitations of this report include its single-case design and the lack of neurological follow-up.

## Conclusions

HSV encephalitis may present with predominantly vestibulocerebellar symptoms and can be complicated by cerebellar infarction and cortical laminar necrosis. Clinicians should maintain a high index of suspicion for central nervous system infection when vertigo occurs in association with fever, altered mental status or seizures. Early MRI and CSF analysis are essential to secure the diagnosis and to detect stroke or other complications. Prompt acyclovir therapy, aggressive seizure management and the careful control of intracranial pressure can lead to meaningful recovery even in severe cases. The recognition of cerebellar CLN on follow-up imaging provides insight into the extent of irreversible cortical injury and may aid prognostication.
